# Rare Pathogenic Variants Predispose to Hepatocellular Carcinoma in Nonalcoholic Fatty Liver Disease

**DOI:** 10.1038/s41598-019-39998-2

**Published:** 2019-03-06

**Authors:** Serena Pelusi, Guido Baselli, Alessandro Pietrelli, Paola Dongiovanni, Benedetta Donati, Misti Vanette McCain, Marica Meroni, Anna Ludovica Fracanzani, Renato Romagnoli, Salvatore Petta, Antonio Grieco, Luca Miele, Giorgio Soardo, Elisabetta Bugianesi, Silvia Fargion, Alessio Aghemo, Roberta D’Ambrosio, Chao Xing, Stefano Romeo, Raffaele De Francesco, Helen Louise Reeves, Luca Vittorio Carlo Valenti

**Affiliations:** 1 Department of Pathophysiology and Transplantation, Università degli Studi di Milano, Fondazione IRCCS Ca’ Granda Ospedale Maggiore Policlinico, Milan, Italy; 2 Internal Medicine and Metabolic Diseases, Fondazione IRCCS Ca’ Granda Ospedale Maggiore Policlinico, Milan, Italy; 3 Northern Institute for Cancer Research, The Medical School, Newcastle University, Newcastle upon Tyne, UK; 4 Department of Surgical Sciences, Liver Transplantation Center, University of Turin, Turin, Italy; 5 Section of Gastroenterology, DIBIMIS, University of Palermo, 90127 Palermo, Italy; 6 Internal Medicine and Gastroenterology Area, Fondazione Policlinico Universitario A. Gemelli, Catholic University of Rome, 00168 Rome, Italy; 7 Clinic of Internal Medicine-Liver Unit, Department of Experimental and Clinical Medical Sciences, University of Udine, Udine, Italy; 8 Division of Gastroenterology, Department of Medical Sciences, University of Torino, Torino, Italy; 9 Division of Gastroenterology and Hepatology Unit, Humanitas Research Hospital and Humanitas University, Rozzano (MI), Italy; 10 “A.M. e A. Migliavacca” Center for the Study of Liver Disease, Division of Gastroenterology and Hepatology, Fondazione IRCCS Ca’ Granda - Ospedale Maggiore Policlinico, Università degli Studi di Milano, Milano, Italy; 11 McDermott Center for Human Growth and Development, University of Texas Southwestern Medical Center, Dallas, TX USA; 12 Sahlgrenska Center for Cardiovascular and Metabolic Research, Wallenberg Laboratory, Cardiology Department, University of Gothenburg, Gothenburg, Sweden; 13 Clinical Nutrition Unit, Department of Medical and Surgical Sciences, Magna Graecia University, Catanzaro, Italy; 14 Istituto Nazionale di Genetica Molecolare (INGM), Romeo ed Enrica Invernizzi, Bioinformatic group, Milan, Italy; 15 Newcastle upon Tyne NHS Foundation Trust, Newcastle upon Tyne, UK; 16 Translational Medicine, Department of Transfusion Medicine and Hepatology, Milan, Italy

## Abstract

Nonalcoholic fatty liver disease (NAFLD) is a rising cause of hepatocellular carcinoma (HCC). We examined whether inherited pathogenic variants in candidate genes (n = 181) were enriched in patients with NAFLD-HCC. To this end, we resequenced peripheral blood DNA of 142 NAFLD-HCC, 59 NAFLD with advanced fibrosis, and 50 controls, and considered 404 healthy individuals from 1000 G. Pathogenic variants were defined according to ClinVar, likely pathogenic as rare variants predicted to alter protein activity. In NAFLD-HCC patients, we detected an enrichment in pathogenic (p = 0.024), and likely pathogenic variants (p = 1.9*10^−6^), particularly in *APOB* (p = 0.047). *APOB* variants were associated with lower circulating triglycerides and higher HDL cholesterol (p < 0.01). A genetic risk score predicted NAFLD-HCC (OR 4.96, 3.29–7.55; p = 5.1*10^−16^), outperforming the diagnostic accuracy of common genetic risk variants, and of clinical risk factors (p < 0.05). In conclusion, rare pathogenic variants in genes involved in liver disease and cancer predisposition are associated with NAFLD-HCC development.

## Introduction

Nonalcoholic fatty liver disease (NAFLD) is now the leading cause of hepatic damage worldwide^1^. In a minority of affected individuals, NAFLD can progress to cirrhosis and hepatocellular carcinoma (NAFLD-HCC), which is emerging as a leading cause of liver-related mortality^2–4^.

Aging, male sex, presence of type 2 diabetes (T2D) and cirrhosis are clinical risk factors for NAFLD-HCC^2^. However, due to the high prevalence of NAFLD in the general population, the low incidence of NAFLD-HCC, but the frequent occurrence of this kind of cancer in non-cirrhotic individuals, and the competition with metabolic comorbidities as a cause of death, mass screening of NAFLD-HCC is currently unfeasible. As a result, most cancers are diagnosed at advanced stages, leading to a dismal prognosis for those affected^5,6^. Therefore, identification of new biomarkers able to improve NAFLD-HCC risk stratification are of paramount clinical importance for the development of targeted and cost-effective surveillance programs.

Heritability is involved in HCC predisposition^7^, and NAFLD has a strong genetic component^8^, as does its progression to advanced disease^9^. A common genetic variation encoding for the I148M variant in *PNPLA3*, the major inherited determinant of hepatic fat accumulation, predisposes to NAFLD-HCC independently of the effect of fibrosis^10,11^. We have also showed that other common genetic variants influencing hepatic fat accumulation, namely those in *TM6SF2* and *MBOAT7*, may improve the ability to discriminate NAFLD patients at risk of HCC^11^. However, possibly because of the still large fraction of missing heritability, carriage of these variants was not specific enough to identify patients at risk of NAFLD-HCC to be implemented in clinical practice^12,13^.

Part of the missing heritability in NAFLD may be accounted for by carriage of rare genetic variants with a large effect size. Indeed, rare germline genetic variants in *TERT* and other loci are associated with occurrence of NAFLD-cirrhosis and familial HCC^14,15^, supporting the hypothesis that rare genetic variants contribute to NAFLD-HCC risk and phenotype variability. However, a systematic evaluation of candidate genes in a large number of affected individuals has not been performed so far.

Taking advantage of next generation whole exome sequencing (WES), the aim of this study was therefore to examine whether variants in candidate genes involved in liver disease and cancer predisposition, that are either pathogenic (that is already linked to a pathological phenotype) or rare and predicted with stringent criteria to alter protein activity (likely pathogenic), are enriched in NAFLD-HCC as compared to healthy individuals and patients with advanced NAFLD. The main outcome was the overall enrichment in pathogenic/likely pathogenic variants, hypothesizing that their identification might be useful in discriminating disease risk and ultimately inform a stratified approach to HCC screening or surveillance in the ever-increasing numbers of patients with NAFLD.

Such an approach enabled us to evaluate a large panel of genes, which would have been not possible by using targeted panels, while restricting at the same time the analysis to those for which there is already solid evidence of a causal relationship with liver disease/cancer.

## Methods

### Study cohorts and design

**Figure 1:**
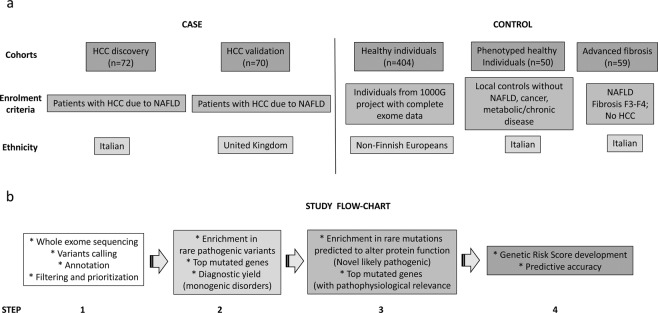
Study design. (**a**) Study cohorts composition and enrolment criteria. (**b**) Study flow-chart. NAFLD: non-alcoholic fatty liver disease. HCC: hepatocellular carcinoma.

The evaluated cohorts and study flow chart are presented in Fig. 1.

The discovery NAFLD-HCC cohort included 72 Italian patients and 70 UK patients, who were enrolled between January 2010 and 2016. All were of Caucasian ancestry.

The diagnosis of HCC was based on the EASL-EORTC clinical practice guidelines for management of hepatocellular carcinoma^16^. Secondary causes of steatosis were excluded on history, including alcohol abuse (≥30 g/day in M/F) and the use of drugs known to precipitate steatosis. Viral and autoimmune hepatitis, hereditary hemochromatosis, Wilson’s disease, overt alpha-1-antitrypsin deficiency and present or previous infection with HBV (HBsAg) and HCV were ruled out using standard clinical and laboratory evaluation as well as liver biopsy features.

Fifty-nine patients with advanced fibrosis due to NAFLD (histological stage F3–F4 or clinically overt cirrhosis) recruited at the Italian institutions during the same period were used as controls. A local ethnically matched control group of comparable sex distribution including 50 Italian healthy blood donors without clinical and biochemical evidences of liver disease, NAFLD, metabolic abnormalities and no alcohol abuse^14^, and the 404 non-Finnish European (NFE) healthy individuals included in the 1000 Genomes database (http://www.internationalgenome.org), for whom complete exome data were publicly available were used as further controls (including 91 Italian and 107 UK individuals).

The study protocol conformed to the ethical guidelines of the 1975 Declaration of Helsinki, was approved by the Ethical committee of the involved Institutions (Fondazione IRCCS Ca’ Granda Policlinico and University of Newcastle upon Tyne), and was performed according to the recommendations of the hospitals involved. Informed consent was obtained from each patient or responsible guardian.

The clinical features of individuals included in the study are presented in Table 1.

**Table 1:** Clinical and genetic features of 251 individuals who underwent whole exome sequencing for evaluation of germline variants in candidate genes involved in liver disease and cancer predisposition.

	HCC discovery (n = 72)	HCC replication (n = 70)	p value (discovery vs. replication)	Advanced fibrosis (n = 59)	Healthy individuals (n = 50)	p value (HCC vs. no-HCC)
Age, years	68 ± 9	74 ± 7	<0.0001	59 ± 10	49 ± 12	<0.0001
Sex, F	17 (24%)	10 (20%)	0.15	21 (36%)	17 (34%)	0.048
BMI, Kg/m^2^	29.8 ± 5.8 (n = 58)	32.7 ± 8.2 (n = 45)	0.12	31.3 ± 4.9 (n = 44)	24.6 ± 2.5 (n = 50)	<0.0001
Type 2 Diabetes, yes	43 (61%)	36 (62%)	0.72	33 (56%)	0	<0.0001
***PNPLA3*** **I148M**						
I/I	18 (25%)	14 (20%)	0.2	13 (22%)	28 (56%)	0.13
I/M	30 (42%)	32 (46%)		26 (44%)	21 (42%)	
M/M	24 (33%)	24 (34%)		20 (34%)	1 (2%)	
***TM6SF2***, **E167K**						
E/E	57 (79%)	53 (76%)	0.84	47 (80%)	38 (76%)	0.59
E/K	14 (20%)	14 (20%)		12 (20%)	12 (24%)	
K/K	1 (1%)	3 (4%)		0	0	
***MBOAT7***, **rs641738 C** > **T**						
C/C	18 (25%)	24 (34%)	0.032	20 (34%)	19 (38%)	0.32
C/T	31 (43%)	38 (54%)		26 (44%)	23 (46%)	
T/T	23 (32%)	8 (12%)		13 (22%)	8 (16%)	

BMI: body mass index; HCC hepatocellular carcinoma; PNPLA3: patatin-like phospholipase domain-containing protein 3; TM6SF2: transmembrane 6 superfamily member 2; MBOAT7: membrane bound O-acyltranferase domain containing 7; GCKR: glucokinase regulatory protein. Data were compared by univariate generalized linear models.

There were four sequential steps to the study (Fig. 1). The first step consisted in whole exome sequencing, variant analysis, identification and prioritization. The second addressed the possibility of enrichment in already known pathogenic variants in candidate genes in NAFLD-HCC cases vs. controls, and identified the most mutated genes and the diagnostic yield for Mendelian monogenic disorders. The third step encompassed the identification of rare variants predicted to alter protein function in the same candidate genes that might be associated with disease predisposition. Finally, a genetic risk score (GRS) for NAFLD-HCC was developed and assessed for its diagnostic accuracy.

### Whole exome sequencing, variants identification, annotation and prioritization

The WES sequencing and analytical pipeline is presented in Supplementary Fig. 1.

Briefly, for the 258 samples included in the EPIDEMIC-NAFLD cohort, which were resequenced for this project (Exome sequencing for the Identification of Inherited Variants Involved in HCC development in NAFLD), DNA was extracted from peripheral blood mononuclear cells, and quantified by a Qubit 2.0 analyzer using the Qubit dsDNA BR Assay Kit (Thermo-Fisher Scientific, Waltham, MA, USA). Samples purity was evaluated using a Nanodrop 1000 spectrophotometer (Thermo-Fisher, Waltham, MA, USA) and integrity was assessed by gel electrophoresis.

DNA libraries were enriched for exome sequencing by the SureSelect Human All Exon v5 kit (Agilent, Cernusco sul Naviglio, Milan, Italy). Sequencing was subsequently performed on the HiSeq4000 platform (Illumina, city). Raw reads quality control was performed using FastQC software (Brabaham bioinformatics, Cambridge, UK). Reads mapping on human GRCh37 genome was performed using MEM algorithm of Burrows Wheeler Aligner (BWA) version 0.7.10^17^. Reads with low quality alignments and duplicate reads were filtered out using Samtools^18^ to generate high quality bam files. Mapping quality control was performed using Picard-tools (http://broadinstitute.github.io/picard) and Bedtools^19^. Sequencing mean depth was of 73x, and no samples exhibit a mean depth lower than 50x (Supplementary Fig. 2 panels a,b). Sequencing resulted in a good target coverage: almost all samples exhibited more than 90% coverage of the target at 20x depth. Importantly, sequencing statistics in terms of input reads, high quality mapped reads, mean depth and coverage, did not show variations among the different cohorts (Supplementary Fig. 2 panel c).

Variant calling was performed following GATK best practices^20^. Briefly, indel local realignment, base quality recalibration and variants calling (Haplotypecaller algorithm) were performed using GATK version 3.3.0^21^. GVCF joint and variants filtering using variant quality score recalibration (VQSR) method were performed. Variants quality score log-odds (VQSLOD) above 99% tranche were considered true positives. To avoid the possibility of calling somatic variants due to the presence of circulating tumor DNA, variants present in <20% of total reads were discarded. Indel left normalization was performed using BCFtools software^22^. Variants annotation was performed using both variant effect predictor (VEP)^23^ and ANNOVAR^24^ tools.

Variants filtering was performed using VCFtools^25^ to exclude variants over VQSLOD threshold and variants which were called in less than 95% of samples. All intronic and synonymous variants according to VEP prediction were excluded from the analyses. Multidimensional scaling of identity-by-state distances analysis was conducted on EPIDEMIC study samples exploiting SNPRelate R Bioconductor package^26^. As shown in Supplementary Fig. 3, in the EPIDEMIC project samples, the first component of variability was explained by the geographic origin of the patients (Italy vs. UK). Whole exome sequencing reads from 1000 genomes project phase 3 (1000 G)^27^ non-Finnish Europeans (NFE; 404 samples) were processed using the same pipeline described for the EPIDEMIC samples.

### Candidate genes selection and classification of variants

Candidate genes were selected according to the literature updated at January 2016, among those whose variants were robustly linked with cancer predisposition syndromes^28^, or mutated in HCC^29^, or predisposing to hereditary liver diseases^30^, or involved in predisposition to telomeres diseases^31^, or in iron and lipid metabolism and NAFLD^32^. The complete list of 181 candidate genes and their classification is presented in the Supplementary File, sheet “Candidate genes”.

For Step 2 (enrichment in rare pathogenic variants in candidate genes and diagnostic rate), variants reported as “likely pathogenic” in the Clinvar (https://www.ncbi.nlm.nih.gov) database^33^, located in candidate genes, and with a minor allele frequency (MAF) <0.05 in 1000 G NFE, ExAC databases and in local healthy controls, were selected.

For Step 3 (enrichment in rare variants predicted to alter protein function, novel likely pathogenic), we used stringent criteria, that is selection of variants determining an alteration of protein sequence (missense, nonsense, splice sites), located in candidate genes, and with a MAF <0.001 in ExAC NFE, MAF <0.005 in the EPIDEMIC project samples, and a CADD Phred >10 (Top 10% of damaging variants)^34^.

### Statistical analysis

For descriptive statistics, continuous variables are shown as mean and standard deviation or median and interquartile range for highly skewed biological variables. Variables with skewed distributions were logarithmically or inverse normally transformed before analyses. All genetic analyses were calculated by using an additive model.

Fisher’s Exact test, multivariate or univariate generalized linear models were used when appropriate. Models were adjusted for clinically relevant covariates, as specified in the Results section. Gene enrichment in rare variants was assessed using the cohort allelic sum test (CAST) approach^35,36^. The association between the frequency of variants in genes significantly enriched in NAFLD-HCC vs. healthy controls was next validated against the cumulative frequency observed in NFE individuals included in the ExAC project (n = 33370) by Fisher’s exact test, adjusted for the number of comparisons. A NAFLD-HCC risk score was developed as previously described^11,37^. The GRS for HCC was calculated by regressing the number of pathogenic/likely pathogenic variant collapsed at the level of single candidate genes and common genetic risk factors for in *PNPLA3*, *TM6SF2* and *MBOAT7* against the presence of HCC. To internally validate the GRS, β coefficients were adjusted using the Jack-knife resampling method. The diagnostic accuracy of different models for NAFLD-HCC prediction was compared by two-sided Venkatraman test^38^. The population attributable risk (PAR) of GRS for NAFLD-HCC was estimated as previously described for case-control studies^39^.

GRS gene functions were explored by pathway enrichment analysis exploiting Ingenuity Pathway Analysis software (Qiagen, Valencia, USA) with default parameters.

Protein features were obtained from Uniprot database (www.uniprot.org) coding variants of interest genes were mapped into reported protein domains and regions. Variants enrichment in protein domains was evaluated by CAST Burden test approach. Lollipop diagrams were generated using Mutation Mapper software (http://www.cbioportal.org/mutation_mapper.jsp).

Statistical analyses were carried out using R statistical analysis software version 3.3.2 (http://www.R-project.org/). P values < 0.05 were considered statistically significant.

## Results

### Pathogenic variants in candidate genes are enriched in NAFLD-HCC

**Figure 2:**
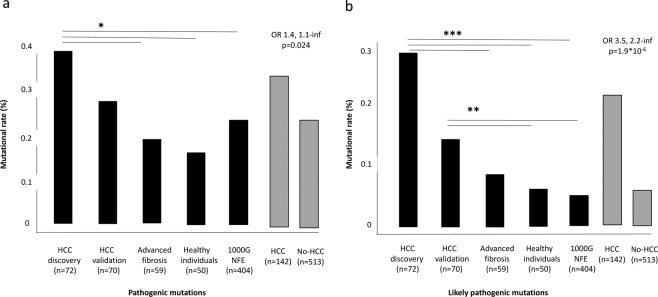
Enrichment in pathogenic variants in patients with NAFLD-HCC. (**a**) Frequency of pathogenic variants (mutational rate %: sum of mutated/total alleles) in NAFLD-HCC cases vs. controls. (**b**) Frequency of likely pathogenic variants (rare variants with high likelihood of altering protein activity) in NAFLD-HCC cases vs. controls. *p < 0.05; **p < 0.01; ***p < 0.005 by Fisher’s exact test.

We first examined pathogenic variants in HCC cases and controls. The list of pathogenic variants identified in candidate genes is presented in the Supplementary File, sheet “Pathogenic”. We identified 68 variants previously linked to pathological phenotypes, which met the inclusion criteria. There was an enrichment in the number of pathogenic variants per individual in candidate genes in NAFLD-HCC patients, as compared to patients with advanced fibrosis, healthy individuals and the 1000 G cohort (Fig. 2a and Supplementary Table 2; OR 1.4, 95% c.i. 1.1-infinite, p = 0.024).

**Figure 3:**
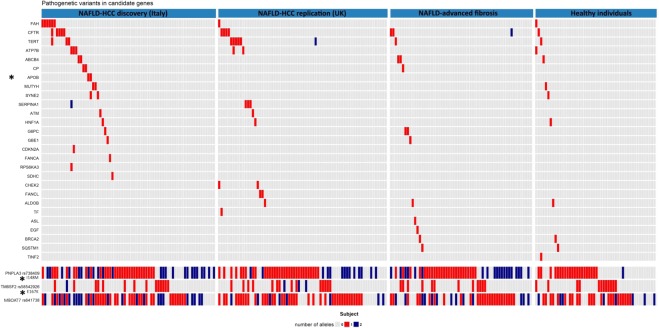
Genes enriched in pathogenic variants. Comutation plot showing the distribution of rare pathogenic variants (upper panel), as well as of common genetic variants (bottom panel) predisposing to hepatic fat accumulation and NAFLD-HCC in the 251 individuals of the EPIDEMIC project. Genes significantly enriched in variants in cases vs. controls are marked by and asterisk (*by Fisher’s exact test). Gene abbreviations are shown in Supplementary Material.

A comutation plot reporting genes interested by pathogenic variants in the newly characterized EPIDEMIC cohorts (excluding 1000 G), as well as common variants previously associated with NAFLD-HCC is reported in Fig. 3. Among the single genes, we found a significant enrichment of variants in the *APOB* gene predisposing to familial hypobetalipoproteinemia (two variants in cases and none in controls, p = 0.047). Furthermore, we confirmed a strong association with the common *PNPLA3* I148M variant (OR 2.49, 95% c.i. 1.89–3.30), and detected an association with the *TM6SF2* E167K variant (OR 1.72, 95% c.i. 1.10–1.24) regulating hepatic lipid compartmentalization. The rs641738 *MBOAT7* variant was associated with NAFLD-HCC in the discovery (OR 1.49, 95% c.i. 1.03–2.15, p = 0.031), but not in the validation cohort (OR 0.81, 95% c.i. 0.56–1.19, p = NS).

After resequencing of candidate genes, according to ClinVar, 19/142 (13.4%) NAFLD-HCC patients vs. 3/59 (5.1%) NAFLD with advanced fibrosis, 3/50 (6.0%) local controls, and 28/404 (6.9%) NFE individuals from 1000 G had a genetic picture consistent with a diagnosis of Mendelian disease predisposing to advanced liver disease or cancer (HCC: 19/142, 13.4%, vs. no-HCC: 34/513, 4.7%; OR 3.15, 95% c.i. 1.57–5.93, p = 0.0005).

### Role of rare variants likely determining an alteration in protein activity

The list of rare variants in candidate genes predicted to alter protein activity (likely pathogenic variants) is provided in the Supplementary File, sheet “likely pathogenic”. We observed an enrichment in rare pathogenic variants in NAFLD-HCC cases vs. controls (Fig. 2b and Supplementary Table 2; OR 3.5 95% c.i. 2.2-inf, p = 1.9*10^−6^). Genes significantly enriched in likely pathogenic variants in NAFLD-HCC cohorts are presented in Table 2. In the overall series or national cohorts, we found a significant enrichment in variants in Telomerase complex genes (*RTEL1*, *TERF2*), DNA and oxidative damage response (*RB1*), and we also highlighted genes involved in regulation of cell growth and proliferation (*STK11*, *TSC1*, *TSC2*, *NF2*, *SMAD4*). Genes involved in regulation of hepatic lipid metabolism, including *APOB* and *MBOAT7* were also enriched in rare likely pathogenic variants, and we detected an enrichment in variants of *SQSTM1*. Most of the associations remained consistent when the cumulative frequency of variants in the candidate genes was compared to that observed in NFE individuals included in the ExAC database. A comutation plot reporting genes significantly enriched in likely pathogenic variants in the EPIDEMIC cohorts is reported in Supplementary Fig. 4.

**Table 2:** Candidate genes significantly enriched in rare variants determining an alteration of protein sequence predicted to alter its function (likely pathogenic mutations) in NAFLD-HCC patients vs. healthy individuals (left panel).

Gene	NAFLD-HCC (n = 72)	Controls (n = 513)	OR	(95% c.i.)	p value*	% carriers NAFLD-HCC	ExAC	OR (95% c.i.)	Adjusted p value**
**Discovery cohort (Italy)**						Replication vs. ExAC NFE			
RTEL1	9	9	7.9	2.7–23.5	7.8*10^–5^	12.5	2.1	5.9 (2.6–11.8)	0.0003
SQSTM1	3	1	22.0	1.7–1161	0.0065	4.2	0.7	5.8 (1.2–18.0)	0.050
TSC2	6	10	4.6	1.3–14.4	0.0083	8.3	3.1	2.7 (0.9–6.1)	0.064
APOB	11	31	2.8	1.2–6.1	0.012	15.3	4.6	3.3 (1.6–6.2)	0.005
TERF2	3	3	7.4	1–55.6	0.027	4.2	0.2	17.4 (3.4–55.0)	0.004
SMAD4	2	1	14.5	1–859	0.041	2.8	0.4	7.4 (0.9–28.3)	0.064
**Validation cohort (UK)**									
STK11	2	1	14.9	1–884	0.039	2.9	0.3	8.4 (1.0–32)	0.051
MBOAT7	2	1	14.9	1–884	0.039	2.9	0.6	4.5 (0.5–17.1)	0.076
NF2	3	4	5.7	1–34.2	0.041	4.3	0.3	12.1 (2.4–37.2)	0.009
RB1	4	8	3.8	1–14.7	0.0201	5.7	1.0	5.9 (1.6–16)	0.018
**Overall**									
RTEL	10	9	4.2	1.5–12	0.0026	7.0	2.1	3.3 (1.5–6.3)	0.006
RB1	7	8	3.3	1–10.5	0.026	4.9	1.0	5.1 (2.0–11)	0.003
TSC1	6	6	3.7	1–14.1	0.027	4.2	1.7	2.5 (0.9–5.6)	0.077
SMAD4	3	1	11.0	1–579	0.034	2.1	3.7	5.6 (1.1–17.2)	0.054
SQSTM1	3	1	11.0	1–579	0.034	2.1	0.7	3.0 (0.6–8.9)	0.086

Significant associations were replicated against NFE individuals included in the ExAC project (n = 33370).

*Evaluated by Burden test. **Evaluated by Fisher’s test adjusted for multiple comparisons. RTEL1: regulator of telomere elongation helicase 1, SQSTM1: sequestosome-1 (p62), TSC2: tuberous sclerosis complex 2, APOB: apolipoprotein B, TERF2: telomere repeat binding factor 2, SMAD4: SMAD (suppressor of mothers against decapentaplegic) family member 4, STK11: serine/threonine kinase 11 (LKB1), MBOAT7: membrane bound O-acyltranferase domain containing 7, NF2: neurofibromin 2, RB1: retinoblastoma 1. Control group was defined as n = 404 NFE individuals form 1000 G project, n = 59 NAFLD patients with advanced fibrosis/cirrhosis, n = 50 local healthy individuals.

In order to check the efficacy of the criteria adopted (frequency and predicted impact) for the identification of likely pathogenic variants, we assessed whether variants in *APOB* (pathogenic/likely pathogenic), which are associated with a clear phenotype that can be assessed by common biochemical tests, influence circulating lipid levels. Results are shown in Supplementary Table 3. Supporting the validity of our selection algorithm, in patients for whom data were available carriage of *APOB* variants was associated with 46% higher HDL cholesterol and 44% lower triglycerides (p = 0.008 and p = 0.001, respectively), consistent with a hypobetalipoproteinemia phenotype.

All in all, these data suggest that rare genetic variants with a very high likelihood of impacting protein activity are associated with the predisposition to develop NAFLD-HCC.

### Genetic risk score development and validation

**Figure 4:**
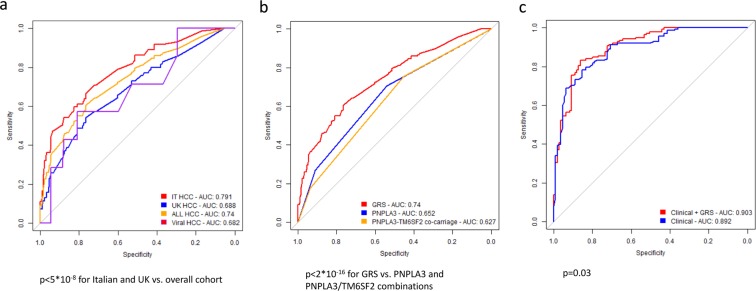
Genetic risk score. Diagnostic accuracy of the Genetic risk score (GRS) for NAFLD-HCC in the 655 individuals included in the study. (**a**) comparison of the diagnostic accuracy in the study cohorts; p < 0.05 for diagnostic accuracy in the overall vs. single cohorts. (**b**) Diagnostic accuracy of GRS vs. *PNPLA3* I148M variant alone and a combination of *PNPLA3* I148M and *TM6SF2* E167K variants in determining NAFLD HCC risk; p < 2*10^−8^ for alternative genetic scores vs. the overall GRS. (**c**) Additive value of adding GRS to a diagnostic model based on clinical risk factors, in determining NAFLD-HCC risk in the 251 individuals of the EPIDEMIC study; p = 0.17. Comparison of diagnostic accuracy was performed by two-sided Venkatraman test.

To examine whether evaluation of common and rare germline genetic variants may be clinically helpful in the stratification of NAFLD-HCC risk, we developed a weighted GRS for this condition and tested its diagnostic accuracy. The GRS coefficients are shown in Supplementary Table 4. In the total cohort of 655 individuals, the GRS was associated with HCC risk (OR 435, 95% c.i. 111–1903; p < 2*10^−16^, OR 4.96, 95% c.i. 3.29–7.55; p = 5.1*10^−16^ for high vs. low GRS). The GRS had an AUROC of 0.74, 95% c.i. 0.69–0.79 for predicting NAFLD-HCC in the total cohort, as compared to 0.79, 95% c.i. 0.73–0.85 and to 0.69, 95% c.i. 0.62–0.75 in the discovery and validation cohorts, respectively (Fig. 4a). The diagnostic thresholds, sensitivity and specificity values are reported in Table S5. The best GRS threshold had a 61% sensitivity and 72% specificity to detect NAFLD-HCC.

The GRS improved the ability to discriminate NAFLD-HCC risk as compared to evaluation of the *PNPLA3* I148M variant alone and of a combination of the *PNPLA3* I148M and *TM6SF2* E167K variants (p < 2*10^−16^; Fig. 4b and Supplementary Table 5).

In the EPIDEMIC cohort with complete data (n = 251), the GRS was associated with NAFLD-HCC (OR 4.96, 95% c.i. 3.29–7.55; p = 5.1*10^−16^), independently of classical risk factors (OR 2.28, 95% c.i. 1.06–4.97, p = 0.04; Table 3). The addition of GRS to a model based on acquired risk factors modified the ability to discriminate NAFLD-HCC (p = 0.03 for comparison of AUC; shown in Fig. 4c). This was mainly due to a slight increase in sensitivity (83% v. 78%, Supplementary Table 6). In the EPIDEMIC cohort, the clinical risk score misclassified 46 patients (19%). Addition of GRS to risk prediction led to a net improvement in classification in 8 individuals (3% of the overall cohort, 17% of misclassified; p = 0.004).

**Table 3:** Independent predictors of NAFLD-HCC in 251 individuals in the EPIDEMIC cohort.

Risk factors	OR	95% c.i.	p value*
GRS, range	59.4	5.3–952	0.002
GRS, high	2.28	1.06–4.97	0.04
Sex, M	1.56	0.64–3.82	0.33
Age, years	1.17	1.12–1.24	4.4*10^–10^
Type 2 diabetes, yes	1.58	0.67–3.67	0.29
Advanced fibrosis, yes	1.60	0.63–4.00	0.31

GRS: Genetic risk score; Range: OR for highest for lowest GRS value in the cohort; High: OR for GRS > 0.22 (median value). *Evaluated at generalized linear model considering covariates shown in the table.

The PAR of the overall panel of genetic variants considered in the GRS for the risk of NAFLD-HCC is presented in Supplementary Table 7. In the overall cohort, the genetic variants considered accounted for 48% (95% c.i. 38–59%) of NAFLD-HCC phenotype variability.

### Characterization of specific variants

The localization of likely pathogenic variants in selected genes (*RETL1*, *APOB*, *SQSTM1*) in individuals with (n = 142) and without (n = 513) HCC is shown in Supplementary Fig. 5a–c. For *APOB*, the rate of truncating variants, determining a higher likelihood of severe impairment in protein function, was higher in HCC vs. non-HCC individuals (p = 0.009). Furthermore, variants in *SQSMT1* tended to localize in a protein region reported to interact with NTRK1 (p = NS).

The summary output of Ingenuity pathway analysis of genes included in the GRS (which were mutated in NAFLD-HCC cases) is presented in Supplementary Table S8. As expected, there was a significant enrichment in pathways related to cancer, hepatocellular carcinoma, liver disease, lipid metabolism, and hereditary disorders. Interestingly, there was an over-representation of genes involved in FXR/RXR activation, and regulation of cell cycle at the G1/S checkpoint.

## Discussion

NAFLD-HCC is an emerging complication of metabolic conditions such as obesity and T2D^2^. Due to the very high prevalence of the population at risk, classic screening strategies are presently unfeasible. Therefore, novel noninvasive biomarkers are urgently needed to improve disease risk stratification. Indeed, although carriage of the common *PNPLA3* I148M variant is a strongly associated with NAFLD-HCC development, taken by itself it is not sufficiently accurate to stratify the risk of this condition^12^.

Here we show that in patients with NAFLD-HCC, pathogenic and likely pathogenic variants in genes linked to liver disease and cancer predisposition are enriched as compared to healthy individuals. Furthermore, we have replicated this result in two independent cohorts.

Although further validation is required before target gene resequencing can be recommended in clinical practice, these findings have potential clinical implications. In the present cohort, resequencing of the candidate genes panel led to the detection of likely predisposing genetic conditions in a large fraction of patients presenting with NAFLD-HCC. This may also aid in the identification of family members for whom screening would be cost effective, or specific preventive treatments may be considered. Furthermore, evaluation of a comprehensive GRS, which takes into consideration rare variants, in individuals with NAFLD may allow a more accurate HCC risk stratification and the implementation of targeted surveillance. Indeed, the comprehensive GRS showed superior diagnostic accuracy as compared to the evaluation of common genetic risk factors, including the *PNPLA3* I148M variant alone^12^, or a combination of *PNPLA3* I148M and *TM6SF2* E167K variants^40^. Furthermore, the GRS improved patient stratification, when considered together with classical risk factors for NAFLD-HCC. The clinical utility of GRS assessment should however be tested in familial and prospective studies evaluating patients with NAFLD and other liver diseases.

Furthermore, the present findings may also have pathophysiological implications worthy of exploration. Firstly, although *PNPLA3* I148M is the major genetic determinant of NAFLD-HCC, no other rare loss-of-function mutations were identified in this gene among the affected patients. This would support the notion that the I148M acquires new functions able to trigger hepatic fat accumulation, alteration of retinol metabolism, and carcinogenesis^41–43^. Secondly, consistently with previous data^11^, other variants favoring hepatocellular fat retention were associated with NAFLD-HCC, including common and rare variants in *TM6SF2* and *MBOAT7* genes. Thirdly, variants in *APOB*, responsible for hypobetalipoproteinemia, were collectively observed in a high proportion of Italian patients (15%), and there was a significant enrichment in pathogenic and truncating mutations in this gene in the overall cohort of NAFLD-HCC patients. *APOB* genetic variants leading to the synthesis of a dysfunctional ApoB100 protein and to a consequent impairment in the export of lipids from hepatocytes within very low-density lipoproteins are responsible for the development of severe hepatic steatosis (hypobetalipoproteinemia, an autosomal dominant disease). At the same time, some *APOB* variants that lead to the alteration of the first portion of the protein result also in altered activity of ApoB48, the protein isoform expressed by enterocytes. This results in retention of chylomicrones, malabsorption of fat and liposoluble vitamins (retinol - vitamin A, vitamin E and vitamin D), known to play a protective role in liver disease progression, and possibly in the alteration of the intestinal barrier. Most importantly, individuals carrying *APOB* mutations had a circulating lipid profile consistent with hypobetalipoproteinemia, providing functional validation of the pathogenicity of the genetic mutations identified. Notably, in line with a causal role of hepatocellular lipid retention in promoting NAFLD-HCC, somatic mutations in *APOB* also frequently occur during hepatic carcinogenesis^44^. The mechanism connecting *APOB* mutations with carcinogenesis is still not completely understood. Induction of hepatocellular lipid accumulation, oxidative stress, and the loss of a possible tumor suppressive activity of APOB are some of the hypothesis that have been raised^45,46^. Therefore, the identification of *APOB* mutations in subjects with NAFLD-HCC would be to allow the diagnosis, in these cases mostly unrecognized, of familial hypobetalipoproteinemia in the first-degree relatives, allowing to establish adequate HCC surveillance.

An additional finding was the novel association between variants in *SQSTM1* and NAFLD-HCC. *SQSMT1* encodes for p62, a component of Mallory-Denk Bodies and hyaline granules^47^. Protein p62 aggregates accumulate in the cytoplasm of damaged liver cells in NASH and HCC^47^, and may promote hepatocytes transformation through the activation of antioxidants and mTOR pathways^48,49^. In keeping, we also identified variants in genes regulating cell growth via the insulin signaling and mTOR pathways, and, in line with previous findings from our group^14,31^, in the telomere regulation machinery. However, as the present study was not designed to this aim, enrichment in variants in specific genes will have to be confirmed in larger cohorts.

This study has some limitations. First, the design was cross-sectional with retrospective data collection, so that GRS for NAFLD-HCC will need to be validated in future prospective studies including individuals with NAFLD and other liver diseases at high baseline risk. However, since HCC remains a relatively rare complication of NAFLD (which affects almost one in three individuals in the general population) the risk of misclassifying healthy controls was minimal, and would have rather led to reduction of the study power. Furthermore, the sample size was relatively limited, and therefore we focused our attention on pathogenic variants in candidate genes, which are already known to cause disease. As we considered healthy individuals from the general population as controls, results are potentially applicable to NAFLD-HCC genetic screening at population level without prior knowledge of liver disease severity status. If it will be proven cost-effective and ethically acceptable, this approach may assist in stratifying the risk of liver disease and HCC, besides of other chronic degenerative diseases. Additional studies are required to discover new variants predisposing to NAFLD-HCC, which were not examined in this study. Moreover, we could not evaluate a control group with advanced fibrosis due to NAFLD for the UK NAFLD-HCC validation cohort, in which a different pattern of mutations was observed as compared to the Italian cohort. Finally, findings may only be applicable to individuals of European descent.

In conclusion, rare pathogenic variants in candidate genes involved in the predisposition to liver disease or cancer are associated with an increased risk of developing NAFLD-HCC.

## Supplementary information

Supplementary material

Dataset 1
