# Supplementary material for: Rare Pathogenic Variants Predispose to Hepatocellular Carcinoma in Nonalcoholic Fatty Liver Disease
Source: Sci Rep. 2019 Mar 6;9:3682. doi: 10.1038/s41598-019-39998-2 (PMC6403344; doi:10.1038/s41598-019-39998-2)
Supplement: Supplementary file 1 — Supplementary material [file 41598_2019_39998_MOESM1_ESM.docx]

**RARE PATHOGENIC VARIANTS PREDISPOSE TO HEPATOCELLULAR CARCINOMA IN NONALCOHOLIC FATTY LIVER DISEASE**

Serena Pelusi ^1, 2^*, Guido Baselli ^1^*, Alessandro Pietrelli ^2^, Paola Dongiovanni ^2^, Benedetta Donati ^1^, Misti Vanette McCain ^3^, Marica Meroni ^1^, Anna Ludovica Fracanzani ^1, 2^, Renato Romagnoli ^4^, Salvatore Petta ^5^, Antonio Grieco ^6^, Luca Miele ^6^, Giorgio Soardo ^7^, Elisabetta Bugianesi ^8^, Silvia Fargion ^1, 2^, Alessio Aghemo^9^, Roberta D’Ambrosio^10^, Chao Xing ^11^, Stefano Romeo ^12,13^, Raffaele De Francesco ^14^, Helen Louise Reeves ^3,15^, Luca Vittorio Carlo Valenti ^1, 2^.

^1^ Department of Pathophysiology and Transplantation, Università degli Studi di Milano, Fondazione IRCCS Ca’ Granda Ospedale Maggiore Policlinico, Milan, Italy.

^2^ Internal Medicine and Metabolic Diseases, Fondazione IRCCS Ca' Granda Ospedale Maggiore Policlinico, Milan, Italy.

^3^ Northern Institute for Cancer Research, The Medical School, Newcastle University, Newcastle upon Tyne, UK.

^4^ Department of Surgical Sciences, Liver Transplantation Center, University of Turin, Turin, Italy.

^5^ Section of Gastroenterology, DIBIMIS, University of Palermo, 90127 Palermo, Italy.

^6^ Internal Medicine and Gastroenterology Area, Fondazione Policlinico Universitario A. Gemelli, Catholic University of Rome, 00168 Rome, Italy.

^7^ Clinic of Internal Medicine-Liver Unit, Department of Experimental and Clinical Medical Sciences, University of Udine, Udine, Italy.

^8^ Division of Gastroenterology, Department of Medical Sciences, University of Torino, Italy.

^9^ Division of Gastroenterology and Hepatology Unit, Humanitas Research Hospital and Humanitas University, Rozzano (MI), Italy

^10^ “A.M. e A. Migliavacca” Center for the Study of Liver Disease, Division of Gastroenterology and Hepatology, Fondazione IRCCS Ca’ Granda - Ospedale Maggiore Policlinico, Università degli Studi di Milano

^11^ McDermott Center for Human Growth and Development, University of Texas Southwestern Medical Center, Dallas, TX

^12^ Sahlgrenska Center for Cardiovascular and Metabolic Research, Wallenberg Laboratory, Cardiology Department, University of Gothenburg, Gothenburg, Sweden.

^13^ Clinical Nutrition Unit, Department of Medical and Surgical Sciences, Magna Graecia University, Catanzaro, Italy.

^14^ Istituto Nazionale di Genetica Molecolare (INGM), Romeo ed Enrica Invernizzi, Bioinformatic group,

Milan, Italy.

^15^ Newcastle upon Tyne NHS Foundation Trust, Newcastle upon Tyne, UK.

*Equal contributors

**SUPPLEMENTARY MATERIAL**

**SUPPLEMENTARY FIGURES LEGENDS**

**Figure S1**. Whole exome sequencing (WES) analytical pipeline. VQSLOD: variants quality score log-odds, HQ-BAM: high quality - binary alignment map, SNP: single nucleotide polymorphism, DB: database, VCF: virtual card file, 1000G: 1000 Genomes project, ExAC: exome aggregation consortium, CADD: combined annotation dependent depletion, ESP: exome sequencing project.

**Figure S2**. **A**) Mean depth of sequencing and **B**) target coverage (depth) for the 251 peripheral DNA samples (discovery NAFLD-HCC n=72, validation NAFLD-HCC n=70, NAFLD-severe fibrosis n=59, healthy individuals n=50) sequenced within the EPIDEMIC-NAFLD project. **C**) Quality control measures in samples stratified by group: hepatocellular carcinoma (HCC), advanced fibrosis, healthy individuals.

**Figure S3**. Multidimensional scaling plot of EPIDEMIC-NAFLD samples (n=251). Common variants, pathogenic variants, and the overall mutational burden are considered.

**Figure S4.** Comutation plot showing the distribution of rare likely pathogenic variants (upper panel) in genes significantly enriched in either single cohorts or the overall cohort, as well as of common genetic mutations (bottom panel) predisposing to hepatic fat accumulation and NAFLD-HCC in the 251 individuals genotyped within the EPIDEMIC project.

**Figure S5**. Localization of likely pathogenic mutations in individuals with (n=142) and without (n=513) HCC. **A**) *RTEL1*, **B**) *APOB*, **C**) *SQSTM1*.

**SUPPLEMENTARY TABLES**

**Table S1.** Clinical features of 7 non cirrhotic patients with HCC associated with HCV infection. Data are expressed as n (%) or mean and (standard deviation), as appropriate.

| Sex, males | 5 (71%) |
| --- | --- |
| Age, years | 70.4 (± 9.4) |
| Steatosis, yes | 3 (42%) |
| Diabetes, yes | 1 (14%) |
| Age at HCC diagnosis | 64.6 (± 5.6) |

**Table S2.** Enrichment of pathogenic and likely pathogenic mutations in NAFLD-HCC cases vs. controls.

| **PATHOGENIC MUTATIONS** |  |  |  |
| --- | --- | --- | --- |
| **Comparison** | **p value** | **OR** | **95% CI** |
| HCC discovery vs. no-HCC | 0.008 | 1.61 | 1.16-Inf |
| HCC validation vs. no-HCC | 0.33 | 1.12 | 0.76-Inf |
| **HCC overall vs no-HCC** | **0.024** | **1.37** | **1.05-Inf** |
| HCC discovery vs. 1000G | 0.017 | 1.53 | 1.10-Inf |
| HCC validation vs. 1000G | 0.43 | 1.06 | 0.72-Inf |
| HCC overall vs. 1000G | 0.055 | 1.30 | 0.99-Inf |
| HCC discovery vs. advanced fibrosis | 0.019 | 1.90 | 1.13-Inf |
| HCC validation vs. advanced fibrosis | 0.24 | 1.32 | 0.75-Inf |
| HCC overall vs. advanced fibrosis | 0.051 | 1.62 | 1.00-Inf |
| HCC discovery vs. healthy individuals | 0.012 | 2.15 | 1.20-Inf |
| HCC validation vs. healthy individuals | 0.16 | 1.49 | 0.80-Inf |
| HCC overall vs. healthy individuals | 0.032 | 1.82 | 1.06-Inf |

| **LIKELY PATHOGENIC MUTATIONS** |  |  |  |
| --- | --- | --- | --- |
| **Comparison** | **p value** | **OR** | **95% CI** |
| HCC discovery vs. no-HCC | 1.0*10^-6^ | 4.61 | 2.74-Inf |
| HCC validation vs. no-HCC | 0.021 | 2.37 | 1.18-Inf |
| **HCC overall vs. no-HCC** | **1.9*10^-6^** | **3.50** | **2.22-Inf** |
| HCC discovery vs. 1000G | 8.0*10^-8^ | 6.25 | 3.48-Inf |
| HCC validation vs. 1000G | 0.005 | 3.21 | 1.52-Inf |
| HCC overall vs. 1000G | 1.3*10^-4^ | 1.88 | 1.41-Inf |
| HCC discovery vs. advanced fibrosis | 0.008 | 3.28 | 1.08-Inf |
| HCC validation vs. advanced fibrosis | 0.032 | 2.50 | 0.69-Inf |
| HCC overall vs. advanced fibrosis | 1.1*10^-7^ | 4.75 | 2.81-Inf |
| HCC discovery vs. healthy individuals | 0.15 | 1.65 | 0.78-Inf |
| HCC validation vs. healthy individuals | 0.73 | 0.85 | 0.35-Inf |
| HCC overall vs. healthy individuals | 0.36 | 1.25 | 0.63-Inf |

OR: odds ratio, 95% c.i.: 95% confidence interval, Inf: infinite.

**Table S3**. Clinical features associated with the presence of APOB pathogenic and likely pathogenic mutations predicted to alter protein function (likely pathogenic) in Italian patients with advanced NAFLD (advanced fibrosis or HCC).

|  | ***APOB*** | | **p value** |
| --- | --- | --- | --- |
|  | **Yes** | **No** |  |
| Age, years | 68.0 ± 6.8  (n=12) | 63.8 ± 10.4  (n=102) | 0.07 |
| Total cholesterol, mg/dl | 158.6 ± 52.4  (n=6) | 183.4 ± 5.7  (n=47) | 0.3 |
| Triglycerides, mg/dl | 80.4 ± 30.7  (n=7) | 142.5 ± 73.7  (n=46) | 0.001 |
| HDL cholesterol, mg/dl | 84.4 ± 26.7  (n=7) | 46.3 ± 19.1  (n=47) | 0.008 |

(): number of individuals for whom data at diagnosis were available.

**Table S4.** Coefficients used to develop the Genetic risk score (GRS) for NAFLD-HCC in the 655 individuals included in the study.

| **variable** | **β Coefficient** | **Jackknifed β Coefficient** | **Bias** | **SD** |
| --- | --- | --- | --- | --- |
| Intercept | 0.072 | 0.072 | -3.12E-06 | 0.001 |
| MTHFR | -0.109 | -0.109 | -5.06E-06 | 0.006 |
| SDHB | -0.080 | -0.080 | 4.59E-06 | 0.001 |
| MUTYH | -0.044 | -0.044 | 1.19E-05 | 0.004 |
| SDHC | 0.668 | 0.668 | 2.30E-06 | 0.002 |
| ATM | 0.420 | 0.420 | -4.15E-05 | 0.016 |
| HNF1A | 0.040 | 0.040 | 1.09E-05 | 0.009 |
| BRCA2 | -0.206 | -0.206 | 3.44E-06 | 0.001 |
| ATP7B | 0.090 | 0.090 | -1.84E-05 | 0.005 |
| TINF2 | -0.234 | -0.234 | -5.23E-06 | 0.004 |
| SYNE2 | -0.085 | -0.085 | -3.49E-05 | 0.003 |
| SERPINA1 | 0.024 | 0.024 | 9.34E-06 | 0.004 |
| FAH | 0.131 | 0.131 | -5.03E-06 | 0.004 |
| FANCA | 0.542 | 0.542 | 1.12E-05 | 0.006 |
| TP53 | -0.090 | -0.090 | 3.13E-05 | 0.004 |
| G6PC | -0.007 | -0.007 | -2.38E-05 | 0.014 |
| APOB | 0.780 | 0.779 | -4.16E-05 | 0.006 |
| FANCL | 0.794 | 0.794 | 3.44E-06 | 0.008 |
| CHEK2 | 0.457 | 0.457 | 1.64E-06 | 0.017 |
| XPC | 0.109 | 0.109 | -6.73E-06 | 0.012 |
| GBE1 | 0.029 | 0.029 | 9.63E-06 | 0.006 |
| TF | 0.321 | 0.321 | 2.12E-06 | 0.013 |
| CP | -0.080 | -0.080 | 4.59E-06 | 0.001 |
| EGF | 0.095 | 0.095 | 9.55E-06 | 0.003 |
| TERT | -0.053 | -0.053 | -1.58E-05 | 0.006 |
| SQSTM1 | -0.368 | -0.368 | 1.25E-05 | 0.006 |
| ASL | 0.014 | 0.014 | 9.59E-06 | 0.004 |
| ABCB4 | 0.099 | 0.099 | 6.75E-06 | 0.003 |
| CFTR | 0.590 | 0.590 | 1.73E-05 | 0.006 |
| CDKN2A | -0.118 | -0.118 | 1.52E-05 | 0.004 |
| ALDOB | 0.790 | 0.790 | 2.85E-06 | 0.010 |
| RPS6KA3 | -0.142 | -0.142 | 1.60E-05 | 0.009 |
| DMD | 0.023 | 0.023 | 0.000143 | 0.006 |
| RB1 | 0.462 | 0.462 | 3.09E-06 | 0.011 |
| SMAD4 | 0.174 | 0.174 | 9.88E-06 | 0.004 |
| RTEL1 | 0.248 | 0.248 | -7.05E-06 | 0.006 |
| TSC1 | 0.103 | 0.103 | 6.74E-06 | 0.002 |
| TM6SF2 | 0.007 | 0.007 | -1.46E-06 | 0.001 |
| MBOAT7 | 0.130 | 0.130 | 4.13E-07 | 0.001 |
| PNPLA3 | 0.109 | 0.109 | -6.73E-06 | 0.012 |

**Table S5.** Diagnostic accuracy of Genetic risk score (GRS) for determining the presence HCC (n=142) as compared to 513 non-HCC individuals considered in the study in the Italian discovery, UK validation, and overall cohort. The comparison with the diagnostic accuracy of the *PNPLA3* I148M variant alone (PNPLA3), and of the combination of the *PNPLA3* I148M and *TM6SF2* E167K variants (PNPLA3+TM6SF2) in the overall cohort is also reported.

| **Cohort** | **AUC** | **95% c.i.** | **Thres** | **95% c.i.** | **Spec** | **95% c.i.** | **Sens** | **95% c.i.** | **NPV** | **95% c.i.** | **PPV** | **95% c.i.** |
| --- | --- | --- | --- | --- | --- | --- | --- | --- | --- | --- | --- | --- |
| **Italy** | 0.79 | 0.73-0.85 | 0.21 | 0.19-0.34 | 0.72 | 0.53-0.95 | 0.71 | 0.46-0.89 | 0.95 | 0.92-0.97 | 0.56 | 0.21-0.6 |
| **UK** | 0.69 | 0.62-0.75 | 0.23 | 0.18-0.33 | 0.77 | 0.46-0.88 | 0.54 | 0.39-0.83 | 0.93 | 0.91-0.96 | 0.24 | 0.17-0.34 |
| **Overall** | 0.74 | 0.69-0.79 | 0.22 | 0.19-0.33 | 0.76 | 0.55-0.89 | 0.61 | 0.46-0.81 | 0.88 | 0.85-0.91 | 0.42 | 0.33-0.55 |
| **PNPLA3** | 0.65 | 0.60-0.70 | - | - | - | - | - | - | - | - | - | - |
| **Het** |  |  | 1 | - | 0.54 | 0.50-0.58 | 0.70 | 0.63-0.77 | 0.82 | 0.80-0.87 | 0.30 | 0.36-0.56 |
| **Hom** |  |  | 2 | - | 0.91 | 0.88-0.94 | 0.27 | 0.20-0.34 | 0.87 | 0.84-0.90 | 0.30 | 0.27-0.33 |
| **PNPLA3+TM6SF2** | 0.63 | 0.61-0.71 | - | - | - | - | - | - | - | - | - | - |
| **Both** |  |  | 2 |  | 0.94 | 0.91-0.96 | 0.18 | 0.11-0.24 | 0.80 | 0.79-0.82 | 0.43 | 0.31-0.56 |
| **Either one** |  |  | 1 |  | 0.45 | 0.41-0.50 | 0.75 | 0.68-0.82 | 0.87 | 0.83-0.90 | 0.28 | 0.25-0.30 |

AUC: area under the curve; Thres: best GRS threshold; Spec: specificity; Sens: sensitivity; NPV: negative predictive value; PPV: positive predictive value; c.i.: confidence interval, Het: heterozygosity, Hom: homozygosity.

**Table S6**. Additive diagnostic accuracy of a HCC genetic risk score based on clinical risk factors (Clinical: age, sex, presence of type 2 diabetes and of advanced fibrosis) plus Genetic risk score (GRS), as compared to clinical factors alone in the 251 individuals of the EPIDEMIC cross-sectional cohort (142 with and 109 without HCC).

|  | **AUC** | **95% c.i.** | **Thres** | **95% c.i.** | **Spec** | **95% c.i.** | **Sens** | **95% c.i.** | **NPV** | **95% c.i.** | **PPV** | **95% c.i.** |
| --- | --- | --- | --- | --- | --- | --- | --- | --- | --- | --- | --- | --- |
| **Clinical + GRS** | 0.90 | 0.86-0.94 | 0.40 | -0.42-0.93 | 0.86 | 0.76-0.94 | 0.83 | 0.74-0.92 | 0.80 | 0.73-0.89 | 0.88 | 0.83-0.95 |
| **Clinical** | 0.89 | 0.84-0.93 | 0.49 | -0.43-1.15 | 0.85 | 0.70-0.97 | 0.78 | 0.67-0.94 | 0.76 | 0.69-0.91 | 0.87 | 0.79-0.97 |

AUC: area under the curve; Thres: best GRS threshold; Spec: specificity; Sens: sensitivity; NPV: negative predictive value; PPV: positive predictive value; c.i.: confidence interval.

**Table S7**. Population attributable risk of NAFLD-HCC in the study cohorts.

| **Overall cohort (including 1000G)** | | | | **EPIDEMIC (unadjusted)** | | | **EPIDEMIC (adjusted*)** | | |
| --- | --- | --- | --- | --- | --- | --- | --- | --- | --- |
|  | **PAR** | **SE** | **95% c.i.** | **PAR** | **SE** | **95% c.i.** | **PAR** | **SE** | **95% c.i.** |
| **Discovery, Italy** | 0.56 | 0.07 | 0.42 - 0.71 | 0.49 | 0.09 | 0.31 - 0.67 | 0.34 | 0.15 | 0.05 - 0.64 |
| **Validation, UK** | 0.40 | 0.08 | 0.24 - 0.56 | 0.3 | 0.11 | 0.08 - 0.52 | 0.1 | 0.22 | -0.32 - 0.53 |
| **Overall** | 0.48 | 0.06 | 0.38 - 0.59 | 0.39 | 0.08 | 0.24 - 0.55 | 0.29 | 0.13 | 0.04 - 0.54 |
| **Non-cirrhotic HCV+** | 0.44 | 0.25 | -0.05 - 0.93 | 0.34 | 0.31 | -0.27 - 0.96 | 0.34 | 0.15 | 0.05 - 0.64 |

SE: standard error; * adjusted for age, sex, presence of type 2 diabetes, and of advanced liver fibrosis F3-F4.

**Table S8 – GRS genes Ingenuity Pathway Analysis summary.** Panel 1 and 2: top pathways among the“canonical pathways” (1) and “tox list” databases (2). Panel 3: networks identified. Panel 4-6: top categories among “disease and disorders” (4), “molecular and cellular functions” (5), and “tox lists:

hepatotoxity” databases (6).

| **1 - Top “canonical pathways”** | |
| --- | --- |
| *pathway* | *pathways p value* |
| Role of BRCA1 in DNA Damage Response | 8.92*10^-11^ |
| Hereditary Breast Cancer Signaling | 2.19*10^-10^ |
| Pancreatic Adenocarcinoma Signaling | 1.78*10^-9^ |
| Ovarian Cancer Signaling | 7.91*10^-9^ |
| Telomerase Signaling | 6.17*10^-8^ |
|  |  |
| **2 - Top “tox lists”** | |
| *list* | *p value* |
| FXR/RXR activation | 8.68*10^-8^ |
| Cell cycle: G1/S checkpoint regulation | 1.38*10^-7^ |
| Liver Proliferation | 2.13*10^-7^ |
| Liver Necrosis/Cell death | 1.21*10^-6^ |
| P53 Signaling | 1.7*10^-6^ |
|  |  |
| **3 - Top networks** | |
| *top network disease and functions* | *Network ID (score)* |
| Metabolic diseases, Cancer, Hereditary disorders | Nw 1 (24) |
| Cancer, Organismal injury and abnormalities, Hereditary disorders | Nw 2 (16) |
| Drug metabolism, Nucleic acid metabolism, Small molecule biochemistry | Nw 3 (16) |
| Protein synthesis, Hematological system development and functions, Inflammatory response | Nw 4 (11) |
| Lipid metabolism, Molecular transport, Small molecule biochemistry | Nw 5 (11) |
|  |  |
| **4 - Top “disease and disorders” categories** | |
| *category* | *disorders p value range* |
| Cancer | 6.67*10^-6^ – 4.34*10^-17^ |
| Hereditary disorders | 4.87*10^-6^ – 4.34*10^-17^ |
| Organismal injury and abnormalities | 6.67*10^-6^ – 4.34*10^-17^ |
| Metabolic disease | 4.24*10^-6^ – 1.47*10^-12^ |
| Gastrointestinal disease | 5.93*10^-6^ – 5.88*10^-12^ |
|  |  |
| **5 - Top “molecular and cellular functions” categories** | |
| *category* | *functions p value range* |
| Cancer | 6.67*10^-6^ – 6.94*10^-14^ |
| Hereditary disorders | 2.68*10^-6^ – 5.55*10^-13^ |
| Organismal injury and abnormalities | 2.62*10^-6^ – 5.55*10^-13^ |
| Metabolic disease | 6.67*10^-6^ – 1.31*10^-12^ |
| Gastrointestinal disease | 2.62*10^-6^ – 1.31*10^-12^ |
|  |  |
| **6 - Top “tox lists: hepatotoxity” categories** | |
| *category* | *lists p value range* |
| Hepatocellular carcinoma | 4.34*10^-2^ – 1.32*10^-9^ |
| Liver Hyperplasia/Hyperproliferation | 1.17*10^-1^ – 1.32*10^-9^ |
| Liver Cirrhosis | 2.28*10^-2^ – 1.07*10^-7^ |
| Liver Proliferation | 2.45*10^-2^ – 1.52*10^-7^ |
| Liver Steatosis | 1.26*10^-1^ – 4.72*10^-7^ |

FXR: farnesoid X receptor; RXR: retinoid X receptor

**SUPPLEMENTARY RESULTS**

**Validation in a pilot group of patients with HCC associated with non-cirrhotic HCV**

To examine whether likely pathogenic variants in candidate genes may predispose to HCC development in other liver diseases, we considered a pilot group of HCV Italian patients, who developed this condition in the absence of advanced liver fibrosis (stage F3-F4). Their clinical features are shown in Supplementary Table 1. The list of pathogenic and likely pathogenic variants identified is reported in the Supplementary File, sheet “Viral non-cirrhotic”. In this pilot cohort, the GRS was significantly associated with HCC risk as compared to healthy controls (OR 4.98, 95% c.i. 3.37-7.41, p=1.3*10^-15^). The AUROC was 0.682 for detecting HCC, comparable to the diagnostic accuracy of the score in the UK NAFLD-HCC cohort (Fig. 4a). The GRS accounted for an estimated 44% of HCC risk variability (Supplementary Table 6).

**Supplementary Legends to Figures**

**Gene abbreviations, related genetic diseases, and inheritance pattern (AD: autosomal dominant, AR: autosomal recessive, XL: X-linked, somatic: usually associated with somatic mutations):** FAH: fumarylacetoacetate hydrolase (tyrosinemia type I, AR); CFTR: cystic fibrosis transmembrane regulator (cystic fibrosis, AR); TERT: telomerase reverse transcriptase (dyskeratosis congenita disease spectrum, AD); ATP7B: copper-transporting ATPase 2 (Wilson’s disease, AR); ABCB4: ATP-binding cassette subfamily B member 4 (MDR3; progressive familial intrahepatic cholestasis type 3, intrahepatic cholestasis of pregnancy, low phospholipid-associated cholelithiasis, AR); CP: ceruloplasmin (aceruloplasminemia); APOB: apolipoprotein B (hypobetalipoproteinemia, AD); MUTYH: MYH glycosylase (familial adenomatous polyposis, AR); SYNE2: spectrin repeat containing nuclear envelope protein 2 (Emery-Dreifuss muscular dystrophy 5, AD & somatic HCC); SERPINA1: Serpina 1 (alpha1-antitrypsin deficiency, AR); ATM: mutated in ataxia-telangiectasia (ataxia-telangiectasia, AR, breast and other cancers); HNF1A: hepatic nuclear factor 1A (Maturity-onset diabetes of the young, type 3, familial hepatic adenomas, renal cell carcinoma, AD); G6PC: glucose-6-phosphatase, catalytic subunit (glycogen storage disease, type Ia – von Gierke’s disease, hepatic adenomas, AR); GBE1: 1,4-alpha-glucan branching enzyme 1 (glycogen storage disease type IV, AR); CDKN2A: cyclin dependent kinase inhibitor 2A (several cancers, somatic & AD); FANCA: Fanconi anemia complementation group A (Fanconi anemia, AR); RPS6KA3: ribosomal protein S6 kinase A3 (Coffin-Lowry syndrome, XL & somatic HCC); SDHC: succinate dehydrogenase complex subunit C (hereditary paraganglioma & pheochromocytoma, and gastrointestinal stromal tumor, AD); CHEK2: checkpoint kinase 2 (Li-Fraumeni syndrome, AD); FANCL: Fanconi anemia complementation group L (Fanconi anemia, AR); ALDOB: aldolase (hereditary fructose intolerance, AR); TF: transferrin (atransferrinemia, AR), ASL: aginosuccinate lyase (arginosuccinic acidura, AR), EGF: epidermal growth factor (hypomagnesemia, type 4, AR), BRCA2: breast cancer type 2 susceptibility protein (breast and other cancers, AD), SQSTM1: Sequestosome-1 (front-temporal degeneration, AD), TINF2: TERF1 interacting nuclear factor 2 (dyskeratosis congenital, AR).

Known common risk variants: PNPLA3: patatin-like phospholipase domain-containing 3; TM6SF2: transmembrane 6 superfamily member 2; MBOAT7: membrane-bound O-acyl transferase 7.
